# Histopathologic features from preoperative biopsies to predict spread through air spaces in early-stage lung adenocarcinoma: a retrospective study

**DOI:** 10.1186/s12885-021-08648-0

**Published:** 2021-08-11

**Authors:** Lanqing Cao, Meng Jia, Ping-Li Sun, Hongwen Gao

**Affiliations:** Department of Pathology, The Second Hospital of Jilin University, 218 Ziqiang Road, Changchun, Jilin, 130041 China

**Keywords:** Histopathologic features, Lung adenocarcinoma, PTNB, Risk stratification, Spread through air spaces

## Abstract

**Background:**

Although spread through air spaces (STAS) is a robust biomarker in surgically resected lung cancer, its application to biopsies is challenging. Moreover, limited resection is not an effective treatment for STAS-positive lung adenocarcinoma. This study aimed to identify histologic features from preoperative percutaneous transthoracic needle biopsies (PTNBs) to predict STAS status in the subsequently resected specimens, and thus help in selecting the surgical extent.

**Methods:**

Between January 2014 and December 2015, 111 PTNB specimens and subsequent resection specimens from consecutive lung adenocarcinoma patients were retrospectively examined. Histopathologic features of PTNB specimens and presence of STAS in subsequent resection specimens were evaluated and correlations between them were analyzed statistically.

**Results:**

The study participants had a mean age of 59 years (range, 35–81) and included 50 men and 61 women. Thirty-six patients were positive for STAS whereas 75 were negative. The micropapillary/solid histologic subtypes of lung adenocarcinoma (26 of 39; 66.7%; *P* <  0.001), necrotic/tumor debris (31 of 42; 73.8%; *P* <  0.001), intratumoral budding (ITB) (20 of 33; 60.6%; *P* <  0.001), desmoplasia (35 of 41; 85.4%; *P* <  0.001), and grade 3 nuclei (12 of 14; 85.7%; *P* <  0.001) were more common in STAS-positive tumors. Micropapillary/solid histologic subtype (OR, 1.35; 95% CI: 1.06, 1.67), ITB (OR, 1.64; 95% CI: 1.09, 2.83), desmoplasia (OR, 1.83; 95% CI: 1.36, 3.12), and N stage (N1 stage: OR, 1.37; 95% CI: 1.19, 1.87) (N2 stage: OR, 1.29; 95% CI: 1.07, 1.73) were independent predictors of STAS.

**Conclusions:**

Micropapillary/solid histologic subtype, ITB, and desmoplasia in preoperative PTNB specimens were independently associated with STAS in the subsequent resection specimens. Therefore, these can predict STAS and may help to optimize therapeutic planning.

## Background

Lung adenocarcinomas (LAC) have a unique pattern of invasion compared to malignancies originating in other organs. Apart from non-lepidic histologic invasion subtypes, infiltrating myofibroblastic stroma, lymphovascular invasion, and pleural invasion, tumor spread through air spaces (STAS), a new distinct invasion concept, was recognized as a pattern of tumor spread in LACs [1]. STAS is defined as micropapillary clusters, solid nests, or single cells beyond the edge of the primary tumor spreading into the air spaces of the surrounding lung parenchyma [1].

STAS is correlated with a considerable reduction in the recurrence-free survival (RFS) and overall survival (OS) in LACs. The presence of STAS has been associated with more aggressive features and poor prognosis in several histological variations of lung cancer. Furthermore, STAS has been recognized as an exclusion criterion for the diagnosis of adenocarcinoma in situ and minimally invasive adenocarcinoma (MIA) [1]. Specifically, STAS is a robust predictor for the local recurrence of early-stage LACs treated with limited resection [2, 3]. Since STAS status can be determined only after the operation to date, it cannot offer substantial assistance for operative decisions. No reliable standard assessment system has been reported for evaluating STAS status by frozen tissue sections during surgical procedures [4]. Thus, determining the potential ability to predict STAS status from preoperative biopsy studies can optimize therapeutic planning for LACs.

In this study, the histologic features of percutaneous transthoracic needle biopsy (PTNB) of LACs were reviewed, and we further correlated the histologic findings of the PTNB specimens with those of the corresponding resected tissues. The main goal of this study was to identify the histologic features that can predict tumor behavior in lung biopsy specimens, thus providing clues for optimal surgical treatment planning.

## Methods

### Patients

**Fig. 1:**
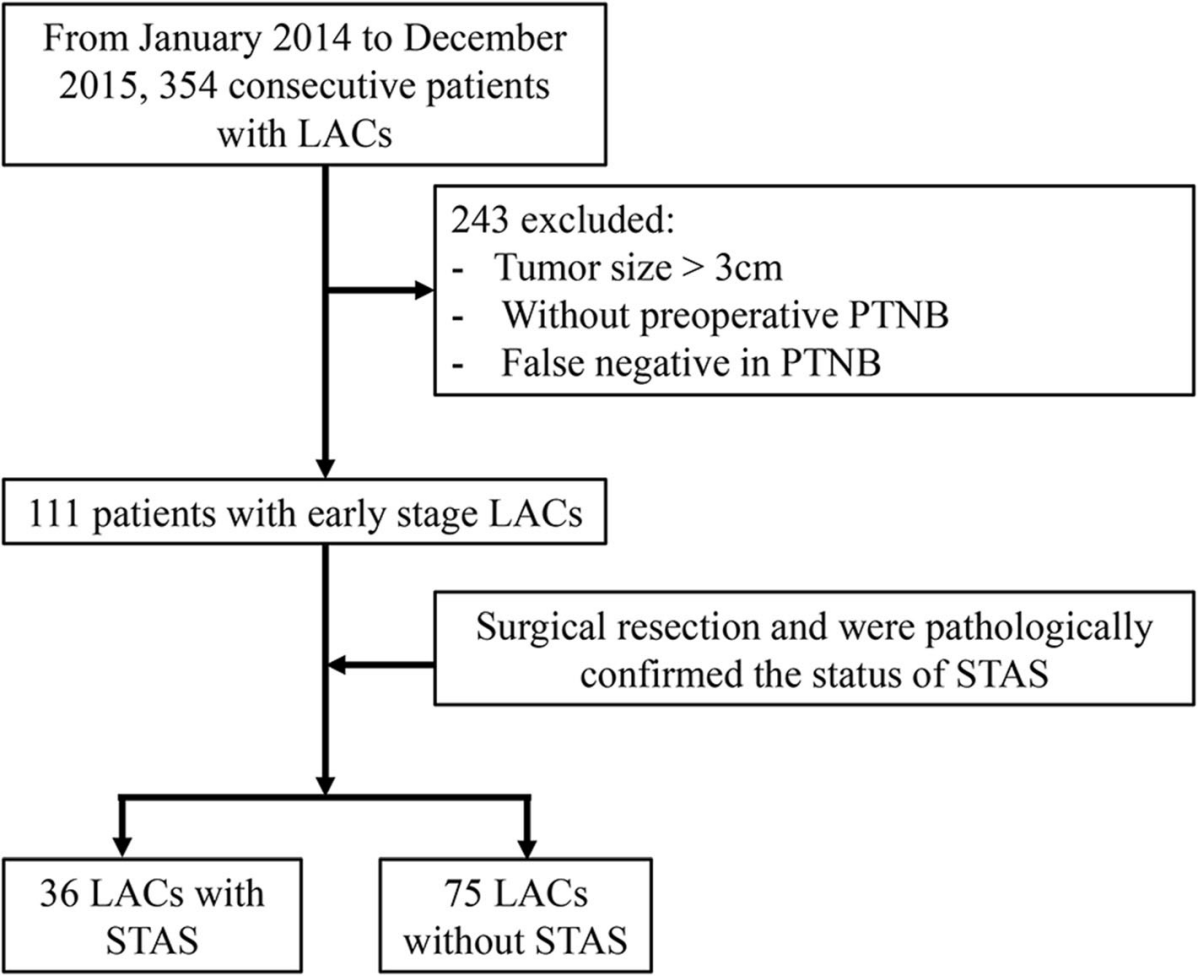
Flowchart of patient inclusion and exclusion

This retrospective study was approved by the Ethics Committee of the Second Hospital of Jilin University (Jilin, China). The requirement for informed consent was waived due to the retrospective nature of the study. Between January 2014 and December 2015, 111 consecutive patients underwent PTNB at our hospital, and were pathologically diagnosed with lung adenocarcinoma. Figure 1 shows patients inclusion and exclusion criteria for the study. All patients underwent a subsequent curative surgical resection. Surgical extent was subclassified as segmentectomy, lobectomy, or pneumonectomy; segmentectomies were collectively referred to as limited resection. Patients were excluded from this study if the pathological findings were inconclusive, or if they had a history of a previous lung operation, neoadjuvant therapy, and specific variants of adenocarcinoma such as invasive mucinous adenocarcinoma, fetal or enteric adenocarcinoma, or other specific accompanying components such as squamous, neuroendocrine, or poor differentiation. Clinical parameters, such as patient age, sex, tumor location, and postoperative outcomes, were collected from the medical records.

### Evaluation of histologic features

Pathological examination of the PTNB and surgical specimens was performed using sequential 3-mm-thick sections stained with hematoxylin and eosin (H&E). Microscopy and imaging were performed using the Olympus BX51 (batch number: 1E39433; filter model: ND6+, ND25+, LBD+, and OP-), Olympus DP26. And the images were acquired by the acquisition software ACDSee Photo Manager 12 at resolution: 2448 × 1920. All tumors were classified according to the latest World Health Organization definitions [1]. All pathological findings were independently evaluated by two single-blinded pathologists. Furthermore, all histological features of PTNB specimens were evaluated by two pathologists blinded to the pathological diagnosis status of subsequent surgical specimens. By definition, STAS was identified as only isolated tumor cells separated from the primary tumor mass and with no direct connection. The mimics and artificial fragments were carefully evaluated and excluded. The following histologic features of PTNB specimens were reviewed: histologic subtype (micropapillary or/and solid vs. others; micropapillary included classical pattern and filigree pattern [5]), intratumoral budding (ITB), necrotic/tumor debris, nuclear grade, desmoplasia, mucin production (extracellular mucin), intracellular mucin, lymphovascular invasion (LVI), and inflammatory reaction. In the subsequent resection specimens, we further evaluated the tumor size and lymph node status. ITB was defined as isolated single cells or a cell cluster composed of < 5 cells. Intraluminal necrotic/tumor debris was defined as tumor cell apoptosis, ghost tumor cells, and neutrophils. Nuclear grade was categorized as 1 (low), 2 (intermediate), or 3 (high). Grade 1 nuclear features resembled the features seen in adenocarcinoma in situ. Grade 3 nuclear features included nuclear enlargement and pleomorphism, and nucleolar prominence. Nuclear grade 2 was defined as nuclear atypia between grade 3 and grade 1 nuclei. The findings were recorded separately.

### Statistical analysis

Categorical and continuous variables representing the clinical and pathologic findings from the positive and negative STAS groups were compared using the Fisher exact test and Mann–Whitney U test. Multivariable logistic regression analyses were conducted to identify the top independent predictors of STAS. Variables with a *p*-value < 0.10 from the univariate analyses were included in the multivariable analyses. All statistical analyses were performed using SPSS statistical software, version 21.0 (SPSS Inc., Chicago, IL, USA).

## Results

### Demographic and Clinicopathologic findings from resection specimens

**Fig. 2:**
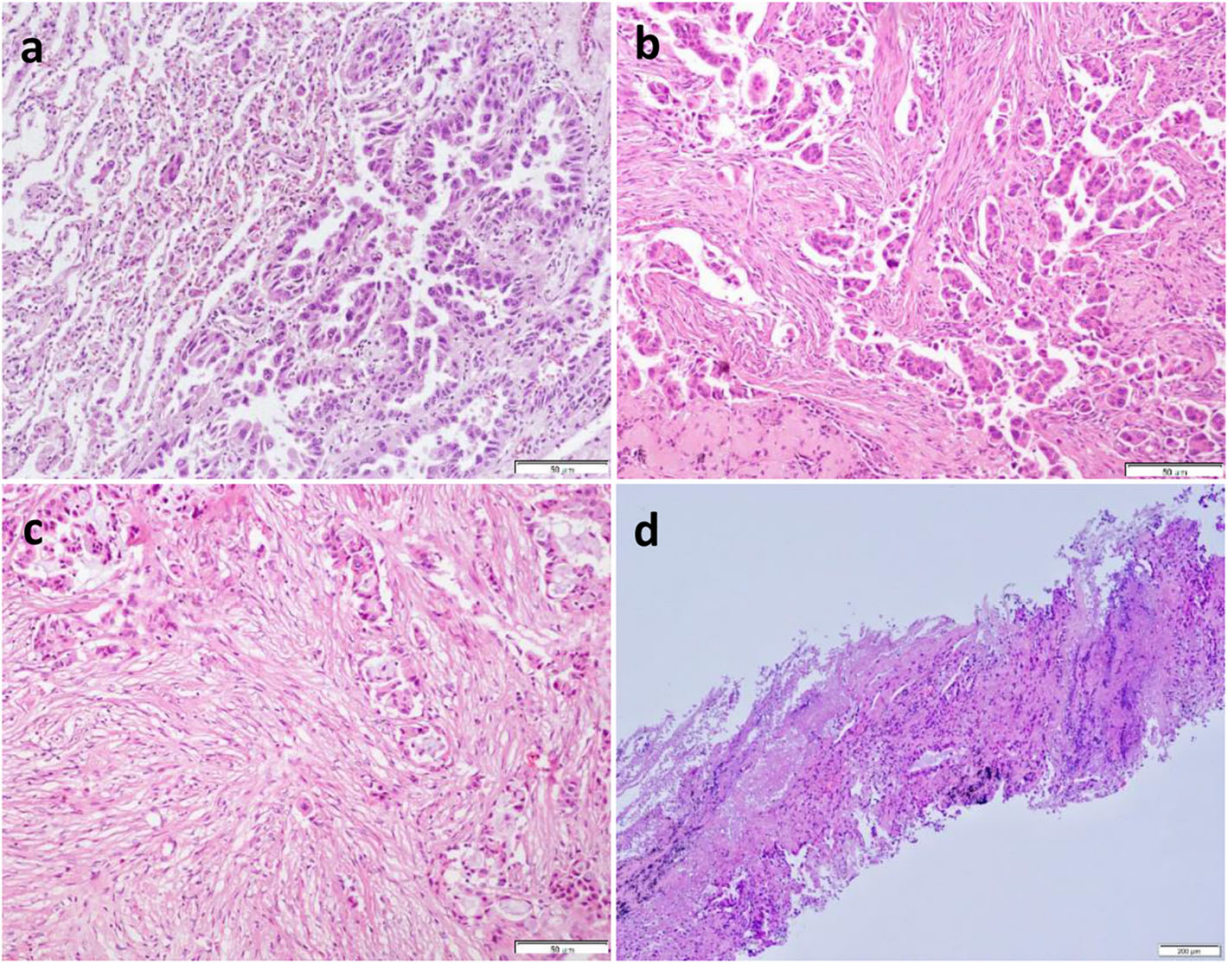
Histological imaging in patients with tumor spread through air spaces (STAS). (**A**) Photomicrograph image showing STAS in a lung adenocarcinoma specimen with STAS in surgical specimens. (**B** and **C**) Photomicrograph images showing micropapillary/solid histologic subtypes of lung adenocarcinoma in PTNB specimens. (**D**) Photomicrograph image showing necrotic/tumor debris in a lung adenocarcinoma with STAS

The demographic and clinicopathologic features of our final study population are shown in Table 1, and an example of LAC with STAS is shown in Fig. 2A**.** The study population had a mean age of 59 years (range, 35–81), and it included 50 men and 61 women. Limited resection was performed in 12.6% (14 of 111) of patients; fewer patients who were positive for STAS (0 of 14; 0%) underwent limited resection than patients who were negative for STAS (36 of 97; 37.1%) (*P* = 0.004).

**Table 1:** Demographics of 111 patients with lung adenocarcinoma who underwent surgical resection

variable		All patients (*n* = 111)	Negative for STAS (*n* = 75)	Positive for STAS (*n* = 36)	*P* value
Age (years)*		59.1 ± 9.1 [35–81]	59.8 ± 9.2 [35–77]	57.6 ± 8.7 [37–81]	0.279
Sex	Female	61	39 (52.0%)	22 (61.1%)	0.419
	Male	50	36 (48.0%)	14 (38.9%)	
Smoking status	Never	71	46 (61.3%)	25 (69.4%)	0.527
	Former or current	40	29 (38.7%)	11 (30.1%)	
Location	Upper & middle lobe	61	38 (50.7%)	23 (63.9%)	0.225
	Lower lobe	50	37 (49.3%)	13 (36.1%)	
Surgery	Lobectomy or pneumonectomy	97	61 (81.3%)	36 (100%)	0.004
	Sublobar resection	14	14 (18.7%)	0 (0.0%)	
Tumor Size (mm)*		22.7 ± 7.9 [8.1–29.5]	22.3 ± 8.4 [8.1–29.5]	23.4 ± 6.6 [8.3–29.1]	0.446
Lymph Node Status	N0	89	65 (86.7%)	24 (66.7%)	0.039
	N1	14	7 (9.3%)	7 (19.4%)	
	N2	8	3 (4.0%)	5 (13.9%)	
Histologic Subtypes	Lepidic	11	11 (14.7%)	0 (0.0%)	0.001
	Acinar	55	46 (61.3%)	9 (25.0%)	
	Papillary	6	2 (2.6%)	4 (11.1%)	
	Micropapillary	16	5 (6.7%)	11 (30.6%)	
	Solid	23	11 (14.7%)	12 (33.3%)	
Lymphovascular Invasion	Absent	91	66 (88.0%)	25 (69.4%)	0.032
	Present	20	9 (22.0%)	11 (30.6%)	
Visceral Pleural Invasion	Absent	101	68 (90.7%)	33 (91.7%)	0.863
	Present	10	7 (9.3%)	3 (8.3%)	

* Data are mean ± standard deviation. STAS, tumor spread through air spaces

The surgical margin was negative in all patients. Among the 111 resection specimens, the main invasive pattern was acinar in 55 (49.5%), papillary in six (5.4%), lepidic in 11 (9.9%), solid in 23 (20.7%), and micropapillary in 16 (14.5%) cases. STAS was more frequently detected in tumors with aggressive pathological characteristic, such as higher N stage (*P* = 0.004), micropapillary/solid histologic subtypes (*P* = 0.001), and LVI (*P* = 0.032).

### Correlation between STAS and histologic features of PTNB specimens

**Fig. 3:**
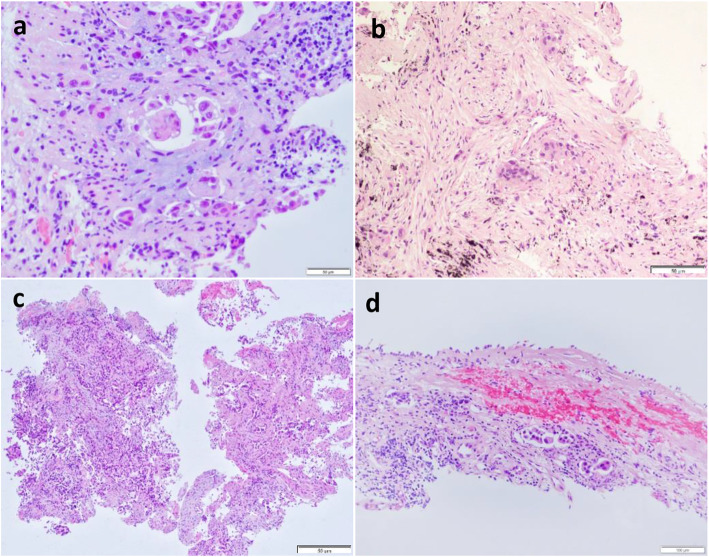
Histological imaging in patients with tumor spread through air spaces (STAS) in PTNB specimens. (**A**) Photomicrograph image showing intratumoral budding in a lung adenocarcinoma with STAS. (**B**) Photomicrograph image showing desmoplasia in a lung adenocarcinoma with STAS. (**C**) Photomicrograph image showing grade 3 nuclei in a lung adenocarcinoma with STAS. (**D**) Photomicrograph image showing lymphovascular invasion in a lung adenocarcinoma with STAS. Note: For better reading, the brightness and contrast were modified in (**B**)

We first evaluated the association between STAS present in the subsequent resection specimens and suspected STAS present in the corresponding PTNB specimens. Only six cases of PTNB with suspected STAS were observed, and no significant correlation with STAS status was found in subsequent surgical specimens. The histologic features of 111 PTNB specimens are summarized in Table 2. A significant relationship was found between the STAS status and the micropapillary/solid histologic subtypes of LAC (26 of 39; 66.7%; *P* <  0.001) (**Fig.** **2****B** and **C**), necrotic/tumor debris (31 of 42; 73.8%; *P* <  0.001) (Fig. 2**D**), ITB (13 of 33; 39.4%; *P* <  0.001) (Fig. 3**A**), desmoplasia (35 of 41; 85.4%; *P* <  0.001) (Fig. 3**B**), and grade 3 nuclei (12 of 14; 85.7%; *P* <  0.001) (Fig. 3**C**). No relationship was found between STAS status and LVI (9 of 19, 47.4%; *P* = 0.127) (Fig. 3**D**), mucinous features (*P* = 0.859), or chronic inflammation (*P* = 0.835) (Table 2).

**Table 2:** Association between spread through air spaces and pathologic variables of the biopsy specimens

variable		All patients	Negative for STAS (*n* = 75)	Positive for STAS (*n* = 36)	*P* value
Histologic subtypes	Others	72	62 (82.7%)	10 (27.8%)	< 0.001
	Micropapillary/Solid	39	13 (17.3%)	26 (72.2%)	
Lymphovascular Invasion	Absent	92	65 (86.7%)	27 (75.0%)	0.127
	Present	19	10 (13.3%)	9 (25.0%)	
Chronic Inflammation	Absent	57	38 (50.7%)	19 (52.8%)	0.835
	Present	54	37 (49.3%)	17 (47.2%)	
Mucinous features	Mucin production	12	7 (58.3%)	5 (62.5%)	0.859
	Cytoplasmic mucin	8	5 (41.7%)	3 (37.5%)	
Necrotic/Tumor Debris	Absent	69	64 (85.3%)	5 (13.9%)	< 0.001
	Present	42	11 (14.7%)	31 (86.1%)	
ITB	Absent	78	62 (82.7%)	16 (44.4%)	< 0.001
	Present	33	13 (17.3%)	20 (55.6%)	
Desmoplasia	Absent	70	69 (92.0%)	1 (2.8%)	< 0.001
	Present	41	6 (8.0%)	35 (97.2%)	
Grade 3 Nuclei	Absent	97	73 (97.3%)	24 (66.7%)	< 0.001
	Present	14	2 (2.7%)	12 (33.3%)	

* Data are mean ± standard deviation. ITB, intratumoral budding; STAS, tumor spread through air spaces

### Histologic predictors of STAS in lung adenocarcinomas

Histologic subtypes (micropapillary/solid vs. others), along with other features with a *P* <  0.10 in univariable analyses, including N stage, necrotic/tumor debris, desmoplasia, grade 3 nuclei, and adenocarcinoma in situ, were included in a multivariable logistic regression analysis (Table 3). Micropapillary/solid histologic subtype (OR, 1.35; 95% CI: 1.06, 1.67), ITB (OR, 1.64; 95% CI: 1.09, 2.83), desmoplasia (OR, 1.83; 95% CI: 1.36, 3.12), and N stage (N1 stage: OR, 1.37; 95% CI: 1.19, 1.87) (N2 stage: OR, 1.29; 95% CI: 1.07, 1.73) were found to be the independent determinants of STAS in the multivariable analysis model.

**Table 3:** Multivariable logistic analysis for predictors of STAS

Variable	Odds Ratio(95% CI)	*P* Value
Histologic subtypes	1.35 (1.06–1.67)	0.016
Necrosis/Tumor Debris	1.03 (0.78–1.24)	0.131
ITB	1.64 (1.09–2.83)	0.011
Desmoplasia	1.83 (1.36–3.12)	< 0.001
Grade 3 Nuclei	1.00 (0.41–1.03)	0.464
N stage	1	
	1.37 (1.19–1.87)	0.013
	1.29 (1.07–1.73)	0.039

CI, confidence intervals; ITB, intratumoral budding; LVI, lymphovascular invasion

## Discussion

This retrospective study quantitatively and systemically evaluated the usefulness of histologic characteristics to predict the presence of STAS in PTNB specimens. Among the various evaluated histologic features, micropapillary/solid histologic subtype, ITB, and desmoplasia were found to be independent predictors of STAS. To the best of our knowledge, this is the first study to assess the role of histologic features of PTNB specimens in predicting STAS as an adverse prognostic indicator in LAC.

The presence of STAS is a powerful independent predictor for poor survival [6–8]. Furthermore, in early-stage LAC with STAS, sublobar/limited resection correlated with a relatively higher risk of recurrence than lobectomy [2, 9–11]. However, no statistical difference was found between lobectomy and sublobar/limited resection in early-stage LAC patients without STAS [12, 13]. These data suggest that an extra wide surgical resection is needed in limited resection of LACs that are positive for STAS; ideally, limited resection should not be considered even in early-stage tumors if findings indicate STAS. Therefore, accurately predicting whether LAC is positive or negative for STAS could assist surgeons in determining which patients are eligible for limited resection, and those who may need an extra lobectomy or postoperative treatment.

PTNB is an effective method for preoperative diagnosis of peripheral lung cancer. However, due to the limited sampling in PTNB, there is a low diagnostic rate or false negative rate in the routine pathology examination. Our data showed a false negative rate for PTNB of 6.5% in LACs less than 3 cm, and of only 2.3% in LACs greater than 3 cm. Therefore, in early-stage lung cancer patients, even if a PTNB is negative, radiology examination results should be combined to determine the subsequent treatment of patients. In our study, the micropapillary/solid histologic subtype was found in 17.3% of STAS-negative cases and in 72.2% of STAS-positive cases. In other studies as well, positive STAS has been predominantly associated with non-lepidic (micropapillary/solid) subtypes [6–8, 14–16]. The micropapillary and solid histologic subtype is associated with aggressive features and indicates poor prognosis; even in patients with stage IA LCAs, a micropapillary and solid prominent growth pattern indicates worse prognosis [7]. Based on our results, we postulate that the presence of a micropapillary and solid growth pattern in PTNB specimens may be a powerful predictor of STAS. Furthermore, our results suggested that ITB and desmoplasia in PTNB could also predict the presence of STAS, independent of other histologic features, including LVI, necrotic/tumor debris, and grade 3 nuclei.

Tumor budding (TB) is an important independent prognostic factor in colorectal cancer and the routine reporting of TB is now advocated using the approach outlined by the International Tumor Budding Consensus Conference guidelines [17]. By definition, STAS was identified as isolated tumor cells separated from the primary tumor mass and with no direct connection to the air spaces; while TB was defined as single tumor cells or small clusters of ≤4 tumor cells at the invasive front stroma. TB has been stratified into peritumoral budding (PTB) and ITB [18]. STAS and TB have special invasion patterns, but it may be difficult to distinguish STAS within air spaces in the alveolar parenchyma beyond the edge of the tumor from TB. However, ITB was located in the tumor center, which could be easily distinguished from STAS; TB was less than 4 cells and almost within the fibrous stroma, while STAS may be beyond 4 cells and primarily within the air spaces. Thus, in most cases, STAS and TB could be clearly distinguished from each another. Tumor dedifferentiation at the invasive fronts shows morphologic features of epithelial-mesenchymal transition (EMT) [19, 20] which is a vital underlying molecular mechanism that enhances the tumor cells’ ability to survive, invade, and disseminate [19]. The cross-talk between tumor cells and tumor stroma favors tumor progression [21]. Therefore, desmoplasia may affect the tumor microenvironment via various cytokines and growth factors that promote EMT, and subsequently, buds and STAS. Supported by our study’s results, ITB and desmoplasia in PTNB are associated with a high ratio of STAS in the correspondent resection specimens. In combination with the contraindication for limited resection in LACs with STAS, these predictive factors can optimize surgical decisions regarding adequate extent of surgery or local ablative therapies.

Our study had several limitations. First, since it was a retrospective single-center study with a relatively small sample size, the statistical power was limited. Second, we only evaluated STAS in LCAs, excluding other histological subtypes of lung cancer, such as invasive mucinous adenocarcinomas, squamous cell carcinomas, or adenosquamous cell carcinomas [22–24]. For a better understanding of STAS, more multi-center studies should be conducted including those on other histologic subtypes rather than adenocarcinoma alone. Finally, our study mainly tries to develop a prediction model; however, the generalizability of our results is unclear. Thus, further validation studies are required.

## Conclusions

In conclusion, STAS is a unique tumor invasion pattern of LAC and a strong prognostic factor in LAC, particularly in cases of limited resection. The micropapillary/solid histologic subtype, ITB, and desmoplasia in PTNB are promising histologic biomarkers for predicting STAS in LACs and may substantially assist the surgeon to optimize the treatment decisions for each patient.

## Data Availability

The datasets generated or analyzed during the current study are available from the corresponding author upon reasonable request.

## Abbreviations

EMT: epithelial-mesenchymal transition
ITB: intratumoral budding
LAC: Lung adenocarcinomas
LVI: lymphovascular invasion
MIA: minimally invasive adenocarcinoma
OS: overall survival
PTNB: percutaneous transthoracic needle biopsies
RFS: recurrence-free survival
STAS: spread through air spaces
